# Two Decades of Teledermatology: Current Status and Integration in National Healthcare Systems

**DOI:** 10.1007/s13671-016-0136-7

**Published:** 2016-03-28

**Authors:** E. Tensen, J. P. van der Heijden, M. W. M. Jaspers, L. Witkamp

**Affiliations:** Department of Medical Informatics, Academic Medical Center, Amsterdam, The Netherlands; KSYOS Health Management Research, Amstelveen, The Netherlands

**Keywords:** Teledermatology, Implementation requirements, Integration national healthcare system, Delivery modalities, Merits

## Abstract

Teledermatology, originating in 1995, has been one of the first telemedicine services to see the light of day. Two decades of teledermatology research is summarized in this review. A literature search was conducted in PubMed. Search terms included “teledermatology,” “teledermoscopy,” “tele wound care,” “telederm*,” “(dermatology OR dermoscopy OR wound care OR skin) AND (telemedicine OR ehealth or mhealth OR telecare OR teledermatology OR teledermoscopy).” Inclusion criteria were (i) Dutch or English written papers and (ii) publication year from 2011 to present or (iii) (systematic) reviews with publication year before 2011. One hundred fourteen publications and 14 (systematic) reviews were included for full text reading. Focus of this review is on the following outcomes: (i) actors (primary, secondary, tertiary), (ii) purposes (consultation, triage, follow-up, education) and subspecialties (tele-wound care, burn care, teledermoscopy (teledermatoscopy), teledermatopathology, and mobile teledermatology), (iii) delivery modalities and technologies (store and forward, real-time interactive, and hybrid modalities using web-based systems, email, mobile phones, tablets, or videoconferencing equipment), (iv) business models, (v) integration of teledermatology into national healthcare systems, (vi) preconditions and requirements for implementation (security, ethical issues, responsibility, reimbursement, user satisfaction, technique, and technology standards), and (vii) added value.

To conclude, teledermatology is an efficient and effective healthcare service compared to in-person care. Teledermatology reduces patients’ travel time and waiting time, avoids (unnecessary) dermatologic visits, and improves access of care to underserved patients.

## Introduction

Telemedicine, as defined by the World Health Organization, is the use of communication technologies in healthcare for the exchange of medical information for diagnosis, treatment, prevention, research, evaluation, and education over a distance (1). Teledermatology is a mature and frequently used form of telemedicine. The first publications about teledermatology listed in PubMed were published in 1995 (2–5) and the number has grown exponentially. At the end of the year 2015, the number of publications in PubMed with search term “teledermatology” evolved to 477 publications.

The visual character of dermatology makes it well-suited for telemedicine. Colors of the skin and distribution of skin lesions provide indications and clues in accurate diagnosing lesions and rashes (6•). Teledermatology has proven to be comparable in accuracy rates to in person conventional care concerning diagnosis, management, and clinical outcomes (7•), clearing many of the barriers mentioned when teledermatology was first implemented. However, some barriers in teledermatology remain, e.g., security, privacy and legal issues, and the absence of palpation of the skin (8, 9), but can be solved relatively easy through selection of patients by the GP, education, and proper implementation of the service.

Teledermatology is currently applied throughout all kinds of medical settings, e.g., in hospital and primary care, nursing homes, home care settings and is applicable in underserved and remote areas to deliver care over a distance. Furthermore, it is applied in countries (e.g., Switzerland, the Netherlands, and the USA) known for their long patient waiting times and/or capacity limits for dermatologic consultation. Teledermatology has been used during wars, in military and maritime settings and reduced the number of medical evacuations (10, 11). Finally, it provides care to patients in developing countries who have no access to (dermatologic) care (12•).

The aim of this narrative review is to give an overview of the current status of teledermatology concerning (1) the actors of teledermatology, (2) the purposes and subspecialties of teledermatology research, (3) the delivery modalities and technologies used, (4) business models used, (5) the integration of teledermatology in national health infrastructures, (6) preconditions and requirements for implementation of teledermatology, and (7) surplus merits of teledermatology.

## Method

A literature search was conducted in PubMed. Search terms included “teledermatology,” “teledermoscopy,” “tele wound care,” “telederm*,” “(dermatology OR dermoscopy OR wound care OR skin) AND (telemedicine OR ehealth or mhealth OR telecare OR teledermatology OR teledermoscopy).” Inclusion criteria were (i) Dutch or English written papers and (ii) publication year from 2011 to present or (iii) (systematic) reviews with publication year before 2011. First, all titles were scanned and all duplicates were removed. Titles that contained “teledermatology” or had a relevant focus were included. Secondly, titles and abstracts were scanned and included if they met the review questions. All papers without an abstract were scanned quickly and were included if they focused on teledermatology. Unavailable publications and publications focusing solely on teledermatopathology were excluded. Finally, one reviewer read all remaining publications and completed a data abstraction form with publication characteristics and relevance for every publication. Results discussed in this review were based on this final selection of the publications and any additional publications that were cited in one of the publications and met the inclusion criteria, but were not in the original search result.

### Search Results

The literature search, as conducted in November 2015, resulted in 787 references and after removal of the duplicates 430 unique publications remained. After title selection, 265 publications were included for abstract selection and 114 publications were included for full text reading. Furthermore, 60 (systematic) reviews, published before 2011 were found and 14 of those reviews remained for full reading after title and abstract selection.

### Actors

**Fig. 1:**
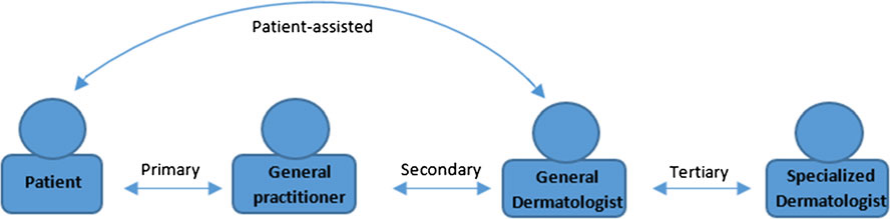
Actors teledermatology

There are different instances of teledermatology in which actors are involved. An overview of different actors in teledermatology is presented in Fig. 1. Primary teledermatology includes direct communication between the patient and the primary healthcare provider (i.e., general practitioner (GP), general nurse) or dermatologists for first diagnosis or referral (6•). Most common is secondary teledermatology. Patients visit the GP and the GP communicates or exchanges medical information of the patient with the dermatologists. Secondary teledermatology is used by primary care providers to receive advice for triage of patient and consults (6•). Other secondary actors who are not explicitly mentioned in the literature are health insurance companies and healthcare institutions, e.g., burn care centers, nursing homes, and emergency departments. Tertiary teledermatology concerns the collaboration and communication among dermatologists (13). Finally, patient-assisted teledermatology is a form of teledermatology in which the patient interacts directly with a healthcare professional, for example in follow-up care in which the patients interacts with a (public health) nurse or wound-care nurse.

### Purposes and Subspecialties of Teledermatology

Pak defines the goal of teledermatology as: “to provide the highest quality of dermatologic care more efficiently by moving patient information rather than patients” (14). Teledermatology can be classified by the different purposes it serves: consultation, triage, follow-up, and education. Likewise, it is used for screening (of melanoma), wound treatment, (international) knowledge exchange between healthcare professionals, second opinion, and referrals prevention.

The systematic review of van der Heijden et al. (13) found different purposes of *tertiary* teledermatology. Tertiary teledermatology could be used for receiving an expert opinion from a more specialized colleague (e.g., academic dermatologist) or a second opinion. Furthermore, it could be used for resident training and ongoing medical education (13).

Teledermatopathology, teledermoscopy (teledermatoscopy), and tele-wound care are subspecialties of teledermatology. Tele-wound care is a subspecialty used in chronic wound care. A study of Litzinger et al. found that 83 % of the nurses improved their productivity and efficiency by using video conferencing in tele-wound care (15). Tele-wound care reduces (transportation and staff) costs, improves quality of life for chronic wound patients, and is equally effective to conventional care (16, 17). Teledermoscopy could be used in the examination of pigmented skin lesions, for the early detection of skin cancer and for triage. Coates et al. summarize an accuracy of teledermoscopic diagnoses, ranging from 75 to 95 % (18). A new application of teledermoscopy concerns the use of mobile teledermoscopy.

Burn care telemedicine makes it possible to get expertise from a healthcare professional of a specialized burn center. It has been shown to be technically and clinically feasible to provide burn care telemedicine (19).

### Delivery Modalities and Technologies

Teledermatology can be delivered by three different modalities: store and forward (SAF), real-time (RT) interactive, and hybrid. Choice of a modality depends on the structure of the local health care system, decisions of stakeholders, like hospital management and physicians as prospective users of the service, payers such as health insurance companies, and the competences of the referring physician (12•). Store and forward is the most offered and used modality in teledermatology (7•, 11, 12•, 20). As described in the literature, the use of store and forward telehealth is increasing and real-time use is decreasing (21).

Table 1 summarizes the advantages and disadvantages of store and forward and real-time interactive technologies. Store and forward and real-time interactive modes of teledermatology have in common that they are independent of space. These modes are especially beneficial in low-resource settings and the USA with large distances between the patient and the dermatologists. By use of these delivery modalities, dermatologists in other geographic areas could be reached (7•, 8, 12•, 22).

**Table 1:** Advantages and disadvantages store and forward and real-time

Store and forward	Real-time interactive
Digital images	Videoconferencing
Asynchronous:	Synchronous:
Space and time independent, flexible	Independent of space, dependent on time, less flexible
No or minimal interaction possible	Direct interaction possible sender and recipient (GP, patient, dermatologist)
Consultation time is short	Time consuming
Low costs	Expensive and not cost-effective short distance
Medical history and images stored and transferred, standardized	More clinical (in depth) and complete information acquired from patient
Response delayed:	Immediate response:
Wait between consultation and advice dermatologist	Advice dermatologist and diagnosis can be obtained immediately during consultation
High resolution digital images	Lower resolution images
Fits better in daily workflow	Interferes with daily workflow

SAF technology includes the exchange of high-quality digital images between a general or nurse practitioner and a dermatologist or between two dermatologists. Distinctive for a store and forward mode is that it can be used time independent, making it more flexible in practice, fitting in daily workflow, and applicable for the exchange of information between different time zones. However, direct interaction between actors is not possible. Use of SAF technology for delivering teledermatology services shortens the consultation time compared to real-time and conventional care, which makes it a lower cost intervention (9). However, responses are delayed, and patients have to wait for the advice of the dermatologist (8, 22).

The real-time (RT) interactive modality uses video conferencing equipment during a teledermatology consultation. Use of this modality makes delivery of teledermatology services location independent but not time independent. RT allows direct interaction between the general practitioner (GP), patient, and dermatologists. They should all be available at the same time, which makes RT consultation scheduling logistically challenging. Furthermore, RT is time consuming, interrupts the routine workflow, and is more expensive (9, 12•, 14, 22). The duration of the videoconference is mostly as long as the conventional consultation and is not cost-effective in case of short travel distances.

Hybrid modalities combine features from SAF and RT. Direct interaction between healthcare professionals and additionally viewing high-quality images is possible by use of hybrid modalities (7•).

Technologies which are used for SAF and RT are web-based systems or email and videoconferencing equipment. Furthermore, mobile phones and tablets could be used for capturing and sending images. Image quality of these devices has been improved and is no longer a barrier in teledermatology (9). Mobile teledermatology and mobile teledermoscopy are specialties that use mobile devices (i.e., phones, tablets) while performing teledermatology. Smartphones and tablets can be used by patients to capture images and transfer them to their healthcare provider or by GP’s to send images for advice to a dermatologist.

### Business Models

Teledermatology has many advantages over current conventional care modes. However, many teledermatology implementations fail when the business models behind the service are either not well understood and subsequently poorly implemented or not implemented at all. Challenges and issues that should be considered in business modeling concern, e.g., technology, security and privacy, legal risks, ethical issues, and reimbursement.

Pak (23) describes five important steps for integrating teledermatology into a well-defined business process and model: “(1) understanding how the organization delivers care, (2) analyzing the alternatives including cost-benefit analysis, (3) obtaining organizational support, (4) formulating an execution plan, (5) training staff and monitoring the process.” Defining a good business and reimbursement model depends on the teledermatology modality used (SAF, real-time, hybrid), consultation, follow-up, and referral process. If teledermatology is implemented in the appropriate setting, it could increase the access and quality of care while decreasing costs (23).

A survey (2011) among teledermatology programs in the USA (20) concluded that 12 of the surveyed programs (33 %) accepted payments from Medicare, Medicaid, Health Maintenance Organizations (HMO), private payers, and self-payers. Furthermore, eight teledermatology programs (22 %) received federal funding from the Veterans Administration or US military and two programs (6 %) provided teledermatology as a voluntary service. Teledermatology programs were reimbursed by private payers (*N* = 25, 69 %), by self-payers (*N* = 22, 61 %), Medicaid (*N* = 20, 56 %), Medicare (*N* = 19, 53 %), and by HMO (*N* = 17, 47 %). Thirty-nine states in the USA receive some reimbursement for telehealth services provided by Medicaid (24). Reimbursement of other countries and business models were not found in our literature search.

In the Netherlands, teledermatology is fully reimbursed and integrated in the national healthcare system. Teledermatology was introduced during the first 5 years of this century in small pilots together with innovating dermatologists and general practitioners on a regional basis as a part of clinical research, thus building clinical evidence and broad basic support among future users. From 2005, health institutions (e.g., KSYOS TeleMedical Center) that solely focused on providing telemedicine and eHealth services, actively implemented teledermatology in the existing health infrastructure. Pivotal factors in the successful implementation have been the focus on change management among and continuous support of future users in the field. GPs were approached to start with teledermatology when the local dermatologists were already on board and could act as local drivers of this new service. Health workers have been trained and supported during the process of implementation. The health institutions providing teledermatology took full responsibility for the entire process including medical responsibility. They contracted medical specialists and general practitioners as well as health insurers and were responsible for quality control. These parties provided safe and user-friendly transmural electronic health records that facilitated the process of teledermatology. Finally, from 2005, the health insurers have reimbursed teledermatology, leading to further increase of its use. This has led to a steady increase in general practitioners using tele consultation services in various fields (e.g., dermatology, ophthalmology, cardiology, and mental health) from 120 in 2005 to an estimated 5500 in 2015 (60 % of all GP’s in the Netherlands).

### Integration in National Healthcare Systems

In 2009, the eHealth survey of the World Health Organization (WHO) showed that a teledermatology service was established in only 16 % of the 114 responding countries (25).

Less is published about the integration of teledermatology in national healthcare systems. In the beginning of 2012, 37 teledermatology programs were active in the USA (20). Reimbursement is often an obstacle for the implementation of telemedicine into (national) health care systems (26). The Veterans Health Administration (VHA) has designed one of the largest teledermatology programs in the USA (21). Furthermore, teledermatology has been broadly integrated in the Dutch Healthcare system since 2006 and is fully reimbursed. In 2014, more than 12 % of the GP visits in the Netherlands was related to dermatological care (27), and in total 27.2 per 1000 patients in GP practice were referred to a dermatologist (27). KSYOS TeleMedical Center (28) provides specialized tele-medical care in the Netherlands. In 2015, KSYOS TeleMedical Center provided 14,900 teledermatology (store and forward) consultations in which 3421 GPs and 247 dermatologists were involved. Since the introduction of teledermatology in 2006, a total of 130,531 teledermatology consultations have been performed by KSYOS TeleMedical Center in the Netherlands.

### Preconditions and Requirements for Implementation of Teledermatology

Perceived barriers and incentives for implementation of teledermatology services differ for primary care physicians and dermatologists and should be taken into account during the implementation. Equipment costs and management and staff training are implementation barriers as perceived by primary care physicians while medical legal liability, diagnostic reliability, and patient follow-up are barriers for academic dermatologists (29, 30). Both groups are concerned about the financial reimbursement of teledermatology (29, 30). In the Netherlands, lack of reimbursement was not an issue during the initial introduction among innovators. However, for the large-scale implementation that has happened in the Netherlands, reimbursement of dermatologists as well as general practitioners has been pivotal.

Various preconditions and requirements should be considered while implementing a teledermatology program. First of all, an important precondition for teledermatology is assignment of persons responsible for the service as a whole, by extension for (in)correct diagnosing and prescribing the associated treatment, and reflecting on the legal risks (31, 32).

Secondly, there are some legal and ethical issues. If images of patients are sent to the dermatologists, instead of the patient itself, a physical physician-patient relationship does not exist according to regulations in some countries (32). Each country has its own laws and regulations that influence implementation. For example, some states in the USA impose restrictions in providing teledermatology to other states in which the physician is not working and licensed, and in the Netherlands teledermatology between patient and dermatologist is only allowed when it concerns a follow-up consultation and the physician-patient relationship has been established in the first face-to-face consultation. This implied that in order to implement teledermatology in the Netherlands, both general practitioner and dermatologist had to be contracted by the same health institution. Doing so, the patient is seen at least once physically by a health worker, in this case the general practitioner, contracted by the health institution. This is mandatory in the Netherlands in order to be able to receive reimbursement from the health care insurers.

Thirdly, security is an essential requirement for teledermatology implementation. Requirements for a secure teledermatology system described in the literature are privacy, availability, authentication, authorization, storage and network security, data encryption, confidentiality, and non-repudiation (9, 33). Data transmission should be reliable and the system should be continuously available, easily accessible and there should be a reliable and secure computer connection. Furthermore, the technical equipment used for making the pictures and sending the images should operate properly. Patients and health care providers should be authorized and verified by a unique authentication number. Confidential patient data should be protected, encrypted, and encoded by transmission. Additionally, the data flow should be logged and it should be documented which health care provider received which information and when. International Organization for Standardization (ISO) standards, like the ISO/TS 13131:2014 (34) on Telehealth services or the ISO/IEC 27001:2013 (35) on information technology security, can be very useful tools to address these issues.

Fourth, although images delivered through teledermatology provide a lot of information, additional (patient) information and medical history is needed for deciding on final diagnoses or treatments. Firstly, data on some patient demographics (e.g., identification number, name, gender, age etcetera) is required. Furthermore, the patient history (complaints and symptoms, allergies, medication use etcetera) and a description of the skin lesion (color, shape, borders, size, location, surface, number of lesions, distribution, and etcetera) could provide necessary clues (11, 22). A technological barrier concerns the interfacing of the teledermatology application with the existing electronic medical record (32).

Furthermore, user satisfaction often is a barrier in the acceptance of technology and a key factor in the implementation of teledermatology. Orruño et al. (36) developed the teledermatology technology acceptance model (based on the technology acceptance model (TAM) of Davis (37)) and determined which factors affect the intention of physicians to use teledermatology. The teledermatology TAM describes the intention of physicians to use teledermatology and the acceptance of teledermatology in three different contexts: the individual (compatibility of technology, attitude), the technological (perceived usefulness of technology, perceived ease of use of technology and habits), and the organizational (facilitators, subjective norm) factors. Habits, compatibility, facilitators, and subjective norm are additional dimensions to the original TAM. Habits encompass behavior which is now, with the use of teledermatology, automatized (36), e.g., do the individuals feel comfortable with the information and communication technology? The developers of the new teledermatology model found that facilitators (organizational infrastructure, training, and support) significantly influence the intention to use teledermatology (36). Training should include how teledermatology provides access to timely dermatologic care, how physicians should take high-quality images and how to send images securely (38). Especially the organizational context of the teledermatology implementation is very important (36), do individuals believe that this organizational infrastructure provides support to use the system? So, implementation requirements for user acceptance of teledermatology are (1) full and continuous technical support to users, (2) training of physicians (3) and an appropriate organizational infrastructure.

The last important factor which should be considered is the standardization of imaging and equipment of teledermatology services. There are no universal imaging standards developed and implemented in teledermatology yet (39). Therefore, Quigley et al. conducted a systematic review summarizing technology and technique imaging standards for acquiring digital dermatologic images (39).

Technology standards include spatial and color resolution, reproduction ratios, post acquisition image processing, color calibration, compression, image output, image archiving and storage, and image security during transmission and storage. A study (1997) concluded that a resolution of 768 × 512 pixels suits teledermatology purposes as well (40). The American Telemedicine Association’s Practice Guidelines for teledermatology (2008) advised at least 24 bits of color which results in 16,777,216 available colors (41). The most recent American Telemedicine Association guideline (2012) recommended minimal 800 × 600 pixels and preferred a resolution of 1024 × 768 pixels for store and forward teledermatology (42).

Technique standards include environmental conditions (i.e., lighting, background, camera position), patient pose and standard view sets, patient consent, privacy, and confidentiality (39). Environmental conditions affect the quality, appearance, and consistency of images (39). And privacy, security, and confidentiality standards depend on region specific laws and regulations.

### Added Value

One of the benefits of teledermatology is reduction of travel by patients. A systematic review by Wootton et al. summarized 18–94 % (mean 43 %) of travel was avoided by teledermatology (43). Another advantage of teledermatology is the number of dermatologic visits averted and a reduction in unnecessary in-patient visits. A recent review by Whited (7•) summarizes that 13–81 % (average 45.5 %) of dermatologic visits were avoided while using store and forward, and 44.4–82 % (average 61.5 %) of visits were averted with real-time interactive teledermatology. As shown by Eminović et al. (44), teleconsultation reduces the number of unnecessary physical referrals to the dermatologist leading to lower costs and higher efficiency. Furthermore, van der Heijden et al. (45) conclude that teledermatology averts 74 % of physical referrals and leads to an 18 % cost reduction compared to in-patient dermatologic care. Teledermatology improves patient access to dermatologic expertise to patients who were underserved by dermatology care for geographic reasons (12•). It further reduces long patient waiting lists, streamlines patient care, and allows shared decision-making with other physicians. Teledermatology consultation, applied in the right setting, provides care equal to but often more efficient and effective as physical patient care and at least does not negatively influence the quality of care delivered to the patient (45, 46). As described by Landow et al. (47) “teledermatology makes three promises: better, cheaper and faster dermatologic care.”

## Discussion

This narrative literature review of PubMed based on publications selected by one reviewer focused on the actors, purposes, subspecialties, delivery modalities and technologies, business models integration of teledermatology services into national healthcare systems, preconditions and requirements for implementation and added value of teledermatology.

Teledermatology is used by healthcare professionals for consultation of other colleagues, triage, and follow-up of patients and education of more junior healthcare professionals. It enables direct digital communication between the patient and primary health care provider or dermatologist, between general practitioners and dermatologists or among dermatologists. Teledermatology can be delivered by three different modalities: store and forward, real-time interactive, and hybrid. As pointed out in the literature, teledermatology has some advantages and could be beneficial for patient care. Teledermatology reduces patient travel time, avoids unnecessary referrals, lowers costs, and improves efficiency of care. More importantly, teledermatology has proven to be at least equally effective as physical patient care and does not negatively influence the quality of care delivered to the patient.

Despite the benefits of teledermatology, experiences of patients should be taken into account while implementing a teledermatology program. Because of methodological deficiencies in the evidence currently available, satisfactory explanations of the underlying reasons for patient satisfaction or dissatisfaction with telemedicine are not available (48). Besides, reliable and validated instruments to measure satisfaction and quality aspects of teledermatology from a patients’ perspective has not been developed yet (7•). In the Netherlands, the consumer quality index (CQ-index) (49), a standardized method for developing surveys and measuring healthcare quality from the patients’ perspective, was introduced in 2006 in order to promote patient-centered care. To measure the quality of care delivered through telemedicine from a patients’ perspective, we are developing a valid and reliable questionnaire, based on the framework of the CQ-index. The responses on such a validated CQ-index for teleconsultation could be used to enhance the quality of care delivered by telemedicine, give choice information to healthcare consumers, advocacy information for patients and patient organizations to inform their members about the quality of care of telemedicine services. Additionally, the results could be used by different stakeholders: by patients to decide about their healthcare provider; by the public health inspection to measure the quality of care; by the health insurance companies to decide about reimbursement; and by the government to monitor quality of healthcare. Furthermore, as indicated by Whited (50) there is a “research gap” on the effect of teledermatology on patients’ quality of life. Quality of life is an important outcome measure for skin diseases and teledermatology may have a positive effect on quality of life of patients. Patients do, for example, not have to visit the dermatologists physically but can visit their GP nearby. Especially for chronic patients, patient-assisted follow-up care at home avoids traveling to a physician and long appointments during work time. Patients can capture images with their smartphone (when they have time) and send the images to their healthcare provider.

There are yet some issues that should be considered before implementing a teledermatology program, e.g., technology, security and privacy, legal risks, ethical issues, and reimbursement. Teledermatology has been fully reimbursed and integrated in the Netherlands and some states in the USA. Reimbursement has a positive influence on the integration in national health systems and the number of teleconsultations conducted in the Netherlands. However, less is published about the integration of teledermatology in healthcare systems of other countries. Due to its merits, we yet expect teledermatology becoming integrated in more healthcare systems in the future. Nami et al. (51) for example believe that teledermatology will become more and more integrated in national health services and clinical practice as smartphones are integrated in our lives. The number of smartphone users is increasing exponentially and will enable us to perform teledermatology via mobile applications. Therefore, we expect that the number of teledermatology services will increase as well.

There are some shortcomings of this review. First, the search was conducted in one database only (PubMed) and no searches were performed in other databases, e.g., MEDLINE and EMBASE. Secondly, publications were selected and included by one reviewer, only which could have resulted in selection bias.

## Conclusion

In conclusion, teledermatology provides care, which is of similar quality compared to conventional care but often more efficient and effective. It is a promising technique for geographically underserved patients and in countries with long patient wait lists. It reduces costs, wait times, travel time, and the number of unnecessary referrals. In the future, more research is needed on the impact of teledermatology on the quality of life and on validated methods for measuring experiences of patients to ensure that teledermatology services are viewed as beneficial from the patient perspective.
